# Primary Hepatic Mesothelial Cyst: A Rare Entity to Be Considered in the Differential Diagnosis of Neonatal Cystic Lesions

**DOI:** 10.7759/cureus.31089

**Published:** 2022-11-04

**Authors:** Joseph M Gosnell, Jana Dejesus, Lindsay Bigham, Daniel Millian, Kimberley C Brondeel, Ravi Radhakrishnan, Heather L Stevenson

**Affiliations:** 1 Pathology, University of Texas Medical Branch at Galveston, Galveston, USA; 2 General Surgery, University of Texas Medical Branch at Galveston, Galveston, USA; 3 Surgery, University of Texas Medical Branch at Galveston, Galveston, USA; 4 Pediatric Surgery, University of Texas Medical Branch at Galveston, Galveston, USA

**Keywords:** hepatopathology, cd31 negative, wt1, d2-40, primary hepatic mesothelial cyst, neonatal cyst, hepatic cyst differential, pediatric surgery, congenital hepatic cyst, mesothelial cyst

## Abstract

We report two cases of primary hepatic mesothelial cysts in neonates previously identified during perinatal imaging. Both neonatal cases were reimaged in the postnatal period, demonstrating the persistence of these cystic hepatic lesions. In both instances, the decision was made to treat with surgical resection and both patients tolerated the surgery well with no significant postoperative complications. Histopathological examination of these lesions discovered a cuboidal lining that was calretinin and WT1 positive and CD31 negative, indicating the diagnosis of a mesothelial cyst of hepatic origin. These cases bring attention to the broad differential diagnosis of congenital primary hepatic cystic lesions, as well as the diagnostic pathway to confirm a primary hepatic mesothelial cyst.

## Introduction

Mesothelial cysts are congenital lesions that are rarely seen or reported in the literature. These lesions are remnants of previous coelomic structures that persist, sometimes into adulthood. They are characterized grossly by having a cystic appearance, often with thin septae (complete or incomplete). Histologically, the cystic space is lined with flat to round cells of mesothelial origin, which can be confirmed by positive immunohistochemical (IHC) staining for calretinin, WT1 and D2-40, and negativity for CD31 [1].

Mesothelial cysts of hepatic origin or primary hepatic mesothelial cysts have been sporadically reported in the literature in both neonates and adult patients. During this review, 15 previously reported cases in the English and non-English literature were found, most of these were in adults, as this entity is rarely reported in the pediatric population. Mesothelial cysts have been observed in nearly every region of the liver, though there is a distinct bias for the round ligament observed in the reported cases. We present two cases of neonates with previously imaged hepatic cystic lesions that were surgically resected after delivery and were histologically characterized as mesothelial cysts via gross appearance and IHC staining.

## Case presentation

Case 1 

**Figure 1:**
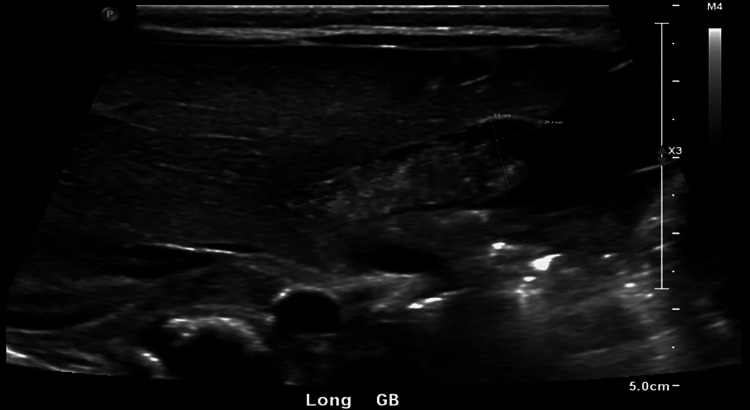
Abdominal ultrasound of cystic lesion (Case 1) Cystic lesion previously identified with intrauterine sonography seen one day post-delivery. The cystic lesion measured 4.8 x 4.7 x 2.6 cm and was observed in the left subhepatic region. The lesion was well-circumscribed with a thin wall, anechoic structure, and without demonstration of internal vascularity. The walls did not demonstrate a sonographic gut signature.

The patient is a five-day-old male born at 36 weeks 5 days gestation to a 39-year-old G6P4 female with little prenatal care. Mother sought prenatal care at 34 weeks, at which time an ultrasound showed a large gestation fetus with a persistent right umbilical vein and an abdominal cystic mass measuring 4.0 x 3.0 x 2.9 cm. The patient was delivered at 36 weeks 5 days with Apgar scores of 7 and 8 at one and five minutes, respectively, but was placed on nasal continuous positive airway pressure and transferred to the neonatal intensive care unit due to respiratory distress. The patient was found to have a moderate-sized Secundum atrial septal defect and patent ductus arteriosus. Laboratory testing showed increased unconjugated bilirubin of 4.5 (normal: 0.1-1.1 U/dL). Repeat ultrasound at one day old showed a well-circumscribed thin-walled anechoic structure without internal vascularity measuring 4.8 x 4.7 x 2.6 cm in the subhepatic region (Figure 1).

Further radiologic evaluation with computed tomography was performed, demonstrating the cystic structure was contiguous with the left lobe of the liver producing a mild mass effect on the stomach. Differential diagnoses included choledochal cyst vs mesenteric cyst vs duplication cyst. Given the cyst’s location and broad differential, the decision was made to proceed with surgical exploration to aid in diagnosis.

Intraoperatively, the patient was noted to have moderate volume ascites. The cyst was arising from the inferior portion of the liver in segments II-IV and excised en bloc. Postoperatively, the patient recovered uneventfully, tolerated full feeds by postoperative day (POD) 2, and was discharged on POD 3. On POD 4, the patient developed serous drainage and mild erythema along the surgical incision concerning possible surgical site infection. Laboratory workup was notable for leukocytosis. No subcutaneous or intra-abdominal fluid collections were demonstrated on imaging. He was started empirically on antibiotics. The erythema and drainage resolved by POD 6.

The antenatal and postnatal abdominal sonograms demonstrated a solitary cystic lesion with thin septae. During resection, it was found that the cystic lesion was in hepatic segments II-IV. After resection, the gross appearance showed a saccular cystic lesion with thin septae, a smooth lining and no cystic contents. Histological examination revealed flat to cuboidal cells lining the cystic space with mesothelial characteristics and no nuclear atypia (Figures 3A, 3B). IHC staining for calretinin and WT1 was positive in the cuboidal cells (Figure 3D), while CD34 was negative, as was CD31 (Figure 3C).

During the third trimester fetal sonogram, the attending physician’s differential diagnosis for the cystic mass observed was either a mesenteric cyst or a dilated loop of the bowel. After delivery, abdominal sonography was concerning for a 4.8 x 2.6 x 4.7 cm mesenteric cyst. The differential for congenital benign cystic lesions of the liver is quite large without considering potential malignant diagnoses. However, a brief list of potential diagnoses includes the following: 1) polycystic liver disease, 2) Caroli disease, 3) biliary hamartoma, 4) ciliated hepatic foregut cyst, 5) mesothelial cyst, 6) choledochal cyst, and 7) duplication cyst [2,3]. When considering potential malignant entities, a clinician must also include 1) undifferentiated embryonal sarcoma and 2) mucinous cystadenoma [3]. 

A hepatic cyst excision was performed during an exploratory laparotomy five days post-delivery. The patient continued to do well post-operatively and was discharged on POD 3. On POD 4, the mother was concerned about the surgical site drainage that soaked a gauze bandage. After treatment with IV antibiotics, the patient was again discharged. Re-check examination on POD 8 showed the patient continues to do well during recovery.

Case 2 

**Figure 2:**
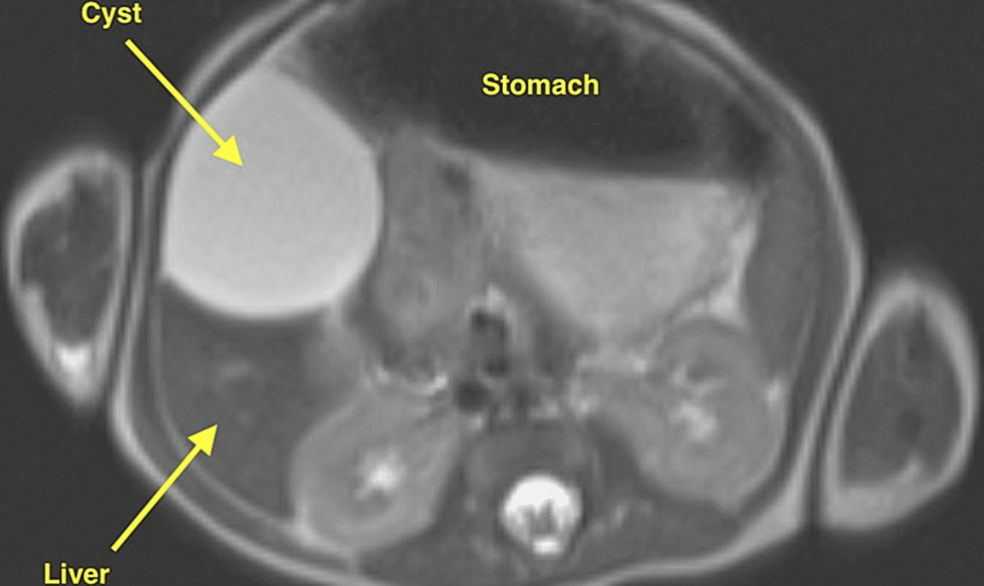
T2-weighted abdominal MRI for Case 2 The hyperintense cystic lesion was measured to be 3.5 x 2.1 x 3.7 cm.

The patient is a 17-day-old female with trisomy 16 mosaicism born at 37 weeks 2 days gestation to a 32-year-old G5P5 female. The patient was delivered with Apgar scores of 3 and 8 at one and five minutes, respectively, but was placed on nasal continuous positive airway pressure and transferred to the neonatal intensive care unit due to respiratory distress. The patient was found to have a complete atrioventricular septal defect with a common AV valve, a large inlet ventricular septal defect, a primum atrial septal defect, a large patent ductus arteriosus with a bidirectional shunt suggestive of increased pulmonary pressure and a small secundum atrial septal defect. Laboratory testing showed an increased AST of 73 U/dL (reference range 13-40). An abdominal MRI revealed a 3.5 x 2.1 x 3.7 cm T2 hyperintense structure (Figure 2) without an internal solid component found in the subhepatic area, abutting both the right hepatic lobe and the gastric antrum. The radiologic evaluation provided a differential diagnosis of gastrointestinal duplication cyst, mesenteric cyst, or exophytic hepatic cyst.

The decision was made to proceed with surgical exploration to aid in diagnosis. The cyst was arising from the liver in segments IV and VIII and excised en bloc. The postoperative course was uneventful, and the patient was discharged on POD 10.

**Figure 3:**
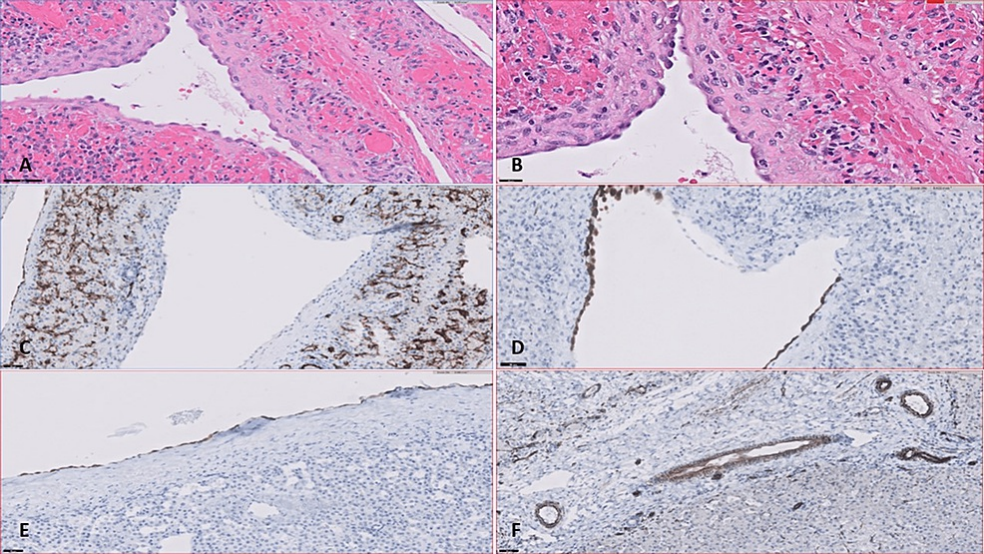
Representative histology from Case 1 and Case 2 Representative sections from Case 1. (A, B) Flat to cuboidal cells with absent nuclear atypia lining the cystic spaces (H&E staining, 400x and 1,000x, respectively). (C) Cells in the cystic lining lack CD31 positivity (100x). (D) Calretinin-positive mesothelial cells in the cystic lining (400x). (E) Calretinin-positive cells lining the cystic space of the Case 2 lesion (400x). (F) WT-1 positivity of these same cells (400x).

The postnatal MRI demonstrated a solitary cystic lesion. At the time of surgical resection, the cyst was identified in hepatic segments IV and VIII. After resection, the gross appearance showed a smooth-walled cystic lesion with a smooth lining and yellow-tinged thin liquid. Histological examination revealed flat to cuboidal cells lining the cystic space with mesothelial characteristics and no nuclear atypia. IHC staining for calretinin and WT-1 was positive in the cuboidal cells (Figures 3E, 3F).

During the postnatal MRI, the attending physician’s differential diagnosis for the cystic mass observed was gastrointestinal duplication cyst, mesenteric cyst, or exophytic hepatic cyst. The differential for congenital benign cystic lesions of the liver is quite large, as discussed in the previous case presentation above.

A hepatic cyst excision was performed during an exploratory laparotomy 17 days post-delivery. The patient continued to do well post-operatively and was discharged on POD 10. Nearly one month after delivery and surgical resection, the patient continues to do well and is being actively managed for the multitude of congenital heart defects consistent with her trisomy 16 mosaicism.

## Discussion

Primary hepatic mesothelial cysts are rare congenital remnants of coelomic structures and can present in unique locations in association with foregut malformations including accessory liver lobes, omphaloceles, and diaphragmatic herniations of hepatic structures. Indeed, after a comprehensive literature review, only 15 previously reported primary hepatic mesothelial cysts were documented, with 10 being reported in adults and five in neonates, often diagnosed in utero (Table 1) [1,3-15].

**Table 1:** Previously reported primary hepatic mesothelial cysts reported in the literature *: not confirmed with IHC NN: neonate

Author(s)	Number of Cases	Patient Age (yrs)	Anatomic Location	Publication Year
Henderson [4]	1*	41	Round Ligament	1909
Dunn et al. [5]	1	51	Couinaud Segment V	1993
Augustine [6]	1	45	Porta Hepatis	2001
Till et al. [7]	1*	0 (5 months)	Superior Liver Surface	2002
Luoma [8]	1*	0 (NN)	Acc. Liver (L Hemithorax)	2003
Rougemont et al. [3]	2	0 (NN)	Accessory Lobe (Oomphalocele)	2007
Lagoudianakis et al. [9]	1*	57	Round Ligament	2008
Komori et al. [1]	1	0 (NN)	L lobe (Oomphalocele)	2008
Jayi et al. [10]	1*	29	Round Ligament	2013
Balaguera et al. [11]	1	42	Round Ligament	2014
van Seventer et al. [12]	1	51	R lobe	2014
Carboni et al. [13]	1	34	Round Ligament	2016
Feo et al. [14]	1	22	Round Ligament	2017
Wang et al. [15]	1	66	L lobe	2021
Total or Average	15	10 Adult, 5 neonate		

The differential diagnosis for congenital hepatic cystic lesions in neonates includes a ciliated hepatic foregut duplication cyst, simple hepatic cyst, biliary hamartoma, Caroli disease, polycystic liver disease, and primary hepatic mesothelial cyst. The list of differential diagnoses grows much larger when considering incidental cystic lesions discovered after birth and can be categorized into four broad categories: 1) developmental, 2) inflammatory, 3) neoplastic, and 4) trauma-related [16].

When determining a diagnosis for a congenital hepatic cyst, the first determining factor is the radiological appearance, either from intra-uterine imaging or ante-natal imaging. Ciliated hepatic foregut cysts are known to occur most often in segment IV and often in a subcapsular location. Simple cysts can appear as either solitary or multiple cysts with an imperceptible wall. Biliary hamartomas appear as multiple irregularly shaped lesions, that may show enhancement on imaging, but no communication with the intrahepatic biliary tree. Caroli disease also appears as multiple cystic lesions with an enhancing central-dot sign on imaging, and communication with the intrahepatic biliary tree, which does not occur with biliary hamartomas. Polycystic liver disease also appears as multiple lesions, though larger in size. Polycystic liver disease also often appears with polycystic kidney disease [16,17]. Finally, mesothelial cysts will appear very similar to simple hepatic cysts, with a very thin wall and as a solitary lesion, usually unilocular in appearance.

When evaluating the histology, ciliated hepatic foregut duplication cysts will be lined with ciliated stratified pseudo-columnar epithelium, mimicking epithelium from the respiratory tract. If the duplication cyst is demonstrating malignant potential, there may be large septations present [18]. Simple hepatic cysts will be lined with cuboidal biliary epithelium and often filled with serous fluid [19]. Biliary hamartomas are also lined with cuboidal biliary epithelium and will possess a sharp, angulated structure to the ducts. Caroli disease cysts will appear as saccular dilations of large intrahepatic bile ducts with a lining similar to both biliary hamartomas and simple hepatic cysts, though this lining will likely be thicker. Polycystic liver disease appears nearly identical to simple hepatic cysts, though there will be multiple of them, and will also have a similar lining to simple hepatic cysts, biliary hamartomas, and Caroli disease lesions [17]. Mesothelial cysts demonstrate a flat to cuboidal lining without nuclear atypia, thin walls with the serous fluid contained inside, and possible thin septations [16].

IHC differentiation becomes difficult, as simple hepatic cysts, biliary hamartomas, Caroli disease, and polycystic liver disease are all biliary duct origin. This means that all will be positive for CK7 and CK19, both characteristic of cells of biliary origin. Both ciliated hepatic foregut duplication cysts and biliary hamartomas are known to be negative for CD-X2, while biliary hamartomas will also be negative for CK20 [16,18]. Ciliated hepatic foregut duplication cysts will also demonstrate positivity for synaptophysin, chromogranin, and calcitonin, due to the neuroendocrine origin cells within the lining of the cyst [20]. Mesothelial cysts, on the other hand, demonstrate positivity for calretinin, D2-40, and WT-1, consistent with the mesothelial origin of the lining, while being negative for CD31 [1]. These four stains are critical to differentiate mesothelial cysts from the lesions of biliary origin that complete the differential diagnosis list but share the simple cystic appearance on imaging and histology (Table 2). 

**Table 2:** Differentiating factors for congenital hepatic lesions

Lesion	Imaging Features	Histological Features	Positive Stains	Negative Stains
Ciliated Foregut Cyst	1. Middle segment location (often segment IV)	1. Lined with ciliated stratified pseudo-columnar epithelium	Synaptophysin	CD-X2
	2. Usually subcapsular	2. May demonstrate large septations	Chromogranin	
	3. Solitary Lesion		Calcitonin	
			Keratin 7	
Simple Hepatic Cyst	1. Appear as either solitary or multiple cysts	1. Lined by cuboidal biliary epithelium	CK7	
	2. Often imperceptibly-thin wall	2. Filled with serous fluid	CK19	
Biliary Hamartoma	1. Multiple irregularly-shaped cysts	1. Dilated small bile ducts, surrounded by dense stroma	CK7	CD-X2
	2. Enhancement may be present	2. Often have sharp, angulated ductal appearance	CK18	CK20
	3. No communication with intrahepatic biliary tree		CK 19	
			Trichrome	
			Low Ki-67	
Caroli Disease	1. Multiple cysts	1. Large intrahepatic bile ducts with saccular dilation	CK7	
	2. "Central dot" sign enhancement	2. Possible to observe communication with benign intrahepatic ducts	CK19	
	3. Communication with intrahepatic biliary tree			
Polycystic Liver Disease	1. Multiple cysts	1. Nearly identical to simple hepatic cysts, but often appear as multiple cysts, either intrahepatic or peribiliary	CK7	
	2. Usually concomitant kidney cysts (polycystic kidney disease)	2. Lined with simple cuboidal biliary epithelium	CK19	
Primary Hepatic Mesothelial Cyst	1. Solitary lesion	1. Lined with flat-to-cuboidal epithelium with no nuclear atypia	Calretinin	CD31
	2. Thin wall	2. Thin wall and filled with serous fluid	D2-40	
	3. Generally unilocular, though septations can appear occasionally	3. Septations may be present	WT-1	

Finally, it is worth noting that in both cases these neonates presented with concomitant congenital cardiac defects. While no publication could be found to support a known connection between primary hepatic mesothelial cysts and congenital cardiac defects, we believe this connection may warrant further investigation in a future study.

## Conclusions

With the increasing use of intrauterine imaging, these entities are discovered in the antenatal and perinatal setting, rather than later in adulthood. When discovered on imaging, the differential is quite wide with both benign and malignant entities as possibilities. However, on histological and IHC evaluation, these entities can be clearly diagnosed due to their mesenchymal appearance and IHC characteristics, such as calretinin and D2-40 positivity with CD31 negativity of the cavity-lining cells. 
